# *De novo* frameshift mutation in ASXL3 in a patient with global developmental delay, microcephaly, and craniofacial anomalies

**DOI:** 10.1186/1755-8794-6-32

**Published:** 2013-09-17

**Authors:** Darrell L Dinwiddie, Sarah E Soden, Carol J Saunders, Neil A Miller, Emily G Farrow, Laurie D Smith, Stephen F Kingsmore

**Affiliations:** 1 Center for Pediatric Genomic Medicine, Children’s Mercy Hospital, Kansas City, MO 64108, USA; 2 Department of Pediatrics, Children’s Mercy Hospital, Kansas City, MO 64108, USA; 3 Department of Pathology, Children’s Mercy Hospital, Kansas City, MO 64108, USA; 4 School of Medicine, University of Missouri-Kansas City, Kansas City, MO 64110, USA; 5 Department of Pediatrics, University of New Mexico Health Science Center, Albuquerque, NM 87131, USA; 6 Clinical Translational Science Center, University of New Mexico, Albuquerque, NM 87131, USA; 7 University of New Mexico, MSC08 4635, Albuquerque, NM 87131-0001, USA

**Keywords:** ASXL3, Bohring-Opitz syndrome, Global developmental delay, Microcephaly, Craniofacial anomalies, *de novo* frameshift, Exome sequencing

## Abstract

**Background:**

Currently, diagnosis of affected individuals with rare genetic disorders can be lengthy and costly, resulting in a diagnostic odyssey and in many patients a definitive molecular diagnosis is never achieved despite extensive clinical investigation. The recent advent and use of genomic medicine has resulted in a paradigm shift in the clinical molecular genetics of rare diseases and has provided insight into the causes of numerous rare genetic conditions. In particular, whole exome and genome sequencing of families has been particularly useful in discovering *de novo* germline mutations as the cause of both rare diseases and complex disorders.

**Case presentation:**

We present a six year old, nonverbal African American female with microcephaly, autism, global developmental delay, and metopic craniosynostosis. Exome sequencing of the patient and her two parents revealed a heterozygous two base pair *de novo* deletion, c.1897_1898delCA, p.Gln633ValfsX13 in *ASXL3*, predicted to result in a frameshift at codon 633 with substitution of a valine for a glutamine and introduction of a premature stop codon.

**Conclusions:**

We provide additional evidence that, truncating and frameshifting mutations in the *ASXL3* gene are the cause of a newly recognized disorder characterized by severe global developmental delay, short stature, microcephaly, and craniofacial anomalies. Furthermore, we expand the knowledge about disease causing mutations and the genotype-phenotype relationships in *ASXL3* and provide evidence that rare, nonsynonymous, damaging mutations are not associated with developmental delay or microcephaly.

## Background

Obtaining a molecular diagnosis for many rare diseases can be an arduous task [1]. The process is often hindered by the rarity of conditions, which is further exacerbated by the clinical heterogeneity, genetic heterogeneity (genocopies) and phenocopies that rare diseases tend to exhibit [2]. Frequently, this results in a process that has been termed the diagnostic odyssey [3]. The NIH Office of Rare Diseases Research reported that it took 1 to 5 years to reach a proper diagnosis for 33% of patients with rare disorders and more than 5 years for 15% of these patients [4]. The recent advent and use of genomic medicine has resulted in a paradigm shift in the clinical molecular genetics of rare diseases – from phenotype-driven diagnosis to genotype-driven diagnosis – and has provided insight into the causes of numerous rare genetic conditions [1]. Briefly genomic medicine, defined as the structured approach to disease discovery, diagnosis, and management that prominently features next-generation sequencing and analysis at a genome scale [5], has provided the impetus for a paradigm shift in the clinical evaluation of rare diseases to identify underlying molecular genetic causes.

Whole exome and genome sequencing of families has been particularly useful in discovering *de novo* germline mutations as the cause of both rare diseases and complex disorders. For example, *de novo* mutations have recently been associated with multiple rare diseases including rare growth disorders characterized by megalencephaly due to mutations in three genes, *AKT3*, *PIK3R2*, *PIK3CA*, [6] and Baraitser-Winter syndrome, characterized by brain malformations due to *de novo* mutations in the actin genes *ACTB* and *ACTG1*[7]. We recently described a *de novo* mutation in *MTOR* as a cause of megalencephaly and intractable seizures (Smith et al., submitted). In addition, new research has implicated *de novo* mutations in the complex disorders of autism [8-10] and schizophrenia [11,12].

First described in 1999, Bohring-Optiz syndrome (BOS) [OMIM, 605039] has recently been shown through the use of next-generation exome sequencing, to be due to *de novo* heterozygous mutations in the additional sex combs-like 1 gene (*ASXL1*) [13]. Two further cases of *de novo* mutations in *ASXL1* in patients with BOS supported the disease-gene association and gave limited insight into genotype-phenotype relationships [14]. Prior to this, the presence of seven of ten features were needed for a clinical diagnosis: trigonocephaly, microcephaly, flammeus nevus, prominent eyes, micro- or retrognathia, abnormal palate, typical BOS posture, feeding difficulties, intrauterine growth restriction (IUGR), and severe/profound learning difficulties [15].

Here, we report on the use of exome sequencing of a proband with considerable overlap with the BOS phenotype and her parents to discover a heterozygous frame shift variant in the additional sex combs-like 3, (*ASXL3*) gene. *ASXL3* is in the same gene family as *ASXL1* and mutations in *ASXL3* appear to be associated with a disorder that is paralogous to BOS.

## Case presentation

**Figure 1:**
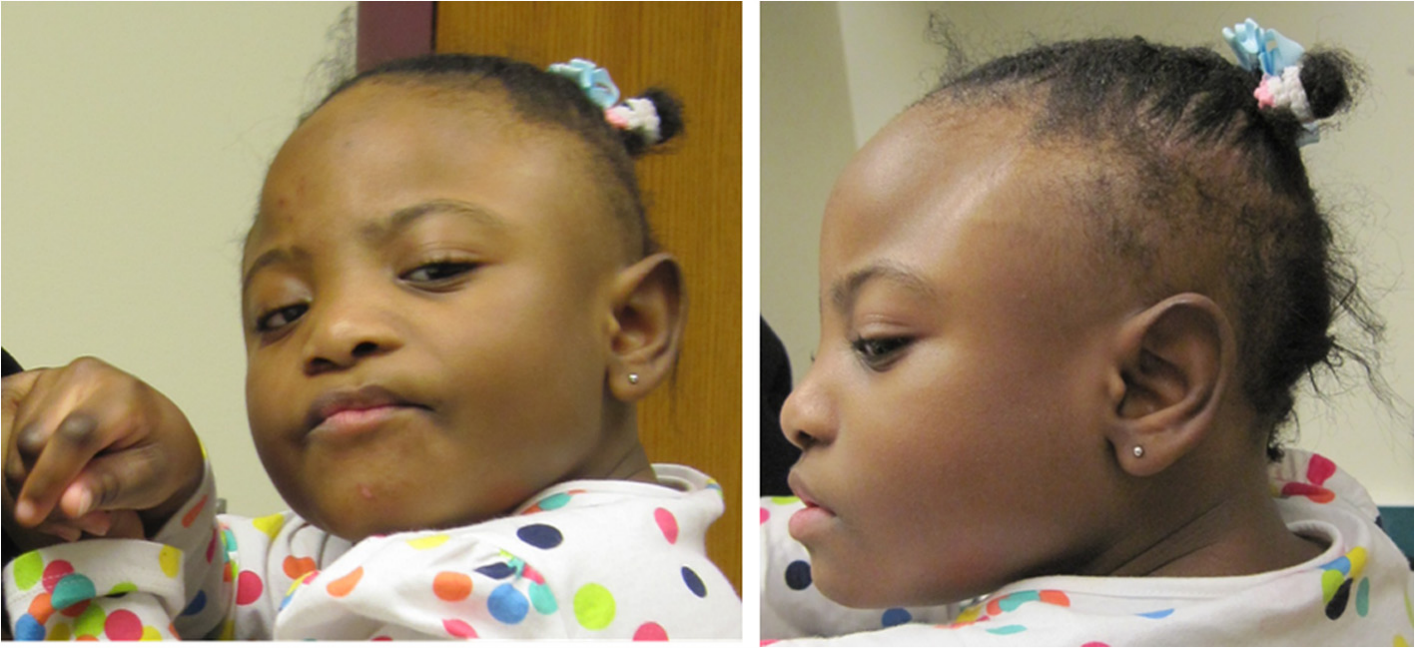
**Patient at 6 years of age.** Note microcephaly, trigoncephaly, hypertelorism, upslanting palpebral fissures, epicanthal folds, periorbital fullness, anteverted nares, and posteriorly rotated ears.

The proband is a 6 year old African American female (CMH000079) with microcephaly, autism, global developmental delay, and metopic craniosynostosis (Figure 1). She was born at 34-weeks gestation following a pregnancy complicated by insulin-dependent diabetes, with maternal blood sugar lability throughout pregnancy. The patient’s mother was a 30 year old gravida 3, para 2 female, with a history of 1 first trimester miscarriage. There was no exposure to alcohol, tobacco, or drugs. Delivery was via cesarean section for breech presentation. At birth the proband had a weight of 1.88 kg (25% for gestational age), length 40.5 cm (3% for gestational age), and occipitofrontal circumference 30 cm (25% for gestational age). Apgar scores were 8 and 9 at one and five minutes, respectively; however, on the first day of life she was transferred to the Neonatal Intensive Care Unit (NICU) for hyperinsulinemic hypoglycemia.

Family history was notable for a maternal half-sister with type 1 diabetes mellitus, who also had a history of hyperinsulinemia and hypoglycemic seizures in infancy, but who has normal cognitive functioning. The mother also had a history of hypoglycemia in childhood that evolved to insulin dependent diabetes mellitus. The mother is otherwise healthy and has no history of learning problems. The patient’s father has hypertension, but is otherwise healthy.

In the first 18 months of life the patient was hospitalized 10 times for vomiting, hypoglycemia, failure to thrive, and episodic irritability. Blood glucose was labile, ranging from 40–215 mg/dL (reference range 60–110 mg/dL). She had two hypoglycemic seizures. A sulfonylurea receptor defect was suspected because of the blood sugar lability in the proband, sister, and mother. Molecular testing of the proband and her mother identified a shared novel variant in the *ABCC8* (ATP-Binding Cassette, Subfamily, Member 8) gene (c. 2143G > A; p.Val115Met) that was considered to be consistent with a diagnosis of Familial Hyperinsulinemic Hypoglycemia Type 1 (HHF1 OMIM #256450) [16,17]. She was therefore placed on diazoxide; a gastrostomy tube and a peripherally inserted central catheter (PICC) line were placed for nutrition and glucose stabilization.

By 18 months, length and weight were between the 10-25th percentiles but head circumference was below the 3rd percentile. She was hypotonic and hyperreflexic. She had dysmorphic facial features including a sloping forehead with metopic ridging, a flat nasal bridge, wide-set eyes and slightly posteriorly rotated ears. The nose had a short columella and hypoplastic alae nasae. Nasolabial folds were smooth and the upper lip had a prominent Cupid’s bow. She had no other dysmorphic features. Developmental milestones were delayed. At 18 months, she could army crawl, had inconsistent visual attention, and was nonverbal. She had episodic irritability with fits of screaming lasting hours, self-injurious behavior, and very poor sleep. She developed repetitive movements, predominately lateral head shaking. Developmental testing demonstrated delay: on the Cognitive Adaptive Test [18], her visual-motor/problem-solving quotient was 33 (mean 100, SD 10); on the Clinical Linguistic and Auditory Milestone Scale [18], language quotient was 42 (mean 100, SD 10). Brain MRI demonstrated mild white matter loss with enlarged lateral ventricles and mild prominence of the sulci. Brain spectroscopy was normal. A head CT confirmed suspected metopic synostosis. Surgical correction of synostosis resulted in less irritability, but did not affect development. An echocardiogram was notable for mild pulmonary artery stenosis, an aortopulmonary collateral vessel, and a small patent foramen ovale. Because of the differences in her development and phenotype from those of her mother and sister, who share the HHF1 diagnosis, additional genetic testing was obtained. Karyotype, microarray, *FMR1* gene analysis, *MECP2* sequencing, and *MECP2* deletion/duplication testing were normal. She remained nonverbal, microcephalic, and globally delayed with repetitive motor behaviors and was diagnosed with autism. She began to walk at around 3 years of age. Through age 6, she continued to be a diagnostic enigma. Several, but not all, of these features have been reported in BOS (Table 1).

**Table 1:** **Comparison of clinical features of CMH000079, patients with ASXL3 mutations described by Bainbridge et al. [**[23]**], and those reported in patients with BOS**

	**CMH000079**	**Reported in Bainbridge et al.**	**Reported in Bohring-Opitz**
**Trigonocephaly**	Y	N	Y
**Microcephaly**	Y	Y	Y
**Flammeus nevus**	N	N	Y
**Prominent eyes**	Y	Y	Y
**Micro- or retrognathia**	N	Y	Y
**Abnormal palate**	N	Y	Y
**Typical BOS posture**	N	N	Y
**Feeding difficulties**	Y	Y	Y
**IUGR**	Y	Y	Y
**Severe/profound learning difficulties**	Y	Y	Y
Upslanting palpebral fissures	Y	NA	Y
Posteriorly rotated eats	Y	Y	Y
High arched palate	N	Y	N
Deep palmar creases	N*	Y	N
Slight ulnar deviation of the hands	N	Y	N
Recurrent infections	Y	NA	Y
Seizures	N	NA	Y
Arrhythmias	N	NA	Y
Apneas	Y#	NA	Y
Epicanthal folds	Y	NA	Y
Broad alveolar ridges	N	NA	Y
Cleft/notch lip	N	NA	Y
Cleft palate	N	NA	Y
Buccal frenulae	N	NA	Y
Depressed nasal bridge	Y	Y	Y
Anteverted nares	Y	Y	Y
Strabismus	N	NA	Y
Anterior chamger abnormalities	N	NA	Y
Myopia	Y	NA	Y
Retinal/optic nerve abnormalities	N	NA	Y
Low hairline	N	NA	Y
Hypertrichosis	Y	NA	Y
Fixed contractures	N	NA	Y
Congenital dislocations	N	NA	Y
Hypotonia	Y (trunk)	Y	Y
Hypertonia	Y (distal)	Y	Y
Brain abnormalities	Y	Y	Y
Genital abnormalities	N	NA	Y
Renal abnormalities	N	NA	Y
Cardiac abnormalities	N	NA	Y

Table adapted from [15], bolded symptoms are the 10 common/specific features of BOS.

NA- Feature not commented on.

*- Some redundancy of skin on hands and palms noted.

#- History of obstructive sleep apnea; no history of central sleep apnea.

At age 6, the proband and her parents were enrolled in an undiagnosed disease program at the Children’s Mercy Hospital in which trios undergo a research exome sequencing study. Briefly, DNA isolated from peripheral blood cells was enriched for all coding exons, UTR, and promoter regions in more than 20,000 characterized genes using the Illumina TruSeq exome enrichment kit and sequenced 2x101 base pairs on an Illumina HiSeq 2000 instrument (Additional file 1: Methods). Alignment and variant characterization were conducted as stated in the Supplement and previously described [19,20]. The patient’s exome was analysed as a trio (two healthy parents and one affected child) for potential mitochondrial [19], *de novo* dominant, and autosomal recessive causes of disease. Zero rare (defined as frequency of 1% or less in dbSNP v137 [21], the 1,000 Genomes Project [22] or CPGM internal variant database [19,20]), likely pathogenic mitochondrial variants were discovered. However, CMH00079 was found to be heterozygous for two apparent *de novo* mutations. A nonsynonymous *de novo* mutation in *VAX1* (c.267C > G, p.Ile89Met) and a two base pair *de novo* deletion, c.1897_1898delCA (p.Gln633ValfsX13) in *ASXL3*, predicted to result in a frameshift at codon 633 with substitution of a valine for a glutamine and introduction of a premature stop codon 13 amino acids downstream were discovered (Additional file 1: Figure S1). The *ASXL3* deletion was seen in 41 of 90 reads covering the region in CMH000079, but not observed in the mother, CMH000080, or father, CMH000081, despite adequate sequence coverage at that nucleotide (Additional file 1: Figure S1). Heterozygous *de novo* truncating variants in *ASXL3* have recently been reported in 4 patients with a novel clinical phenotype similar to Bohring-Opitz syndrome, and are consistent with this patient’s clinical findings (Table 1, [23]). In addition, two, rare, nonsynonymous, compound heterozygous mutations were also discovered in *LYST* (c.597C > G; p.Asp199Glu & c.298C > T; p.Leu100Phe). After expert review the variants in *VAX1* and *LYST* were deemed unlikely to be pathogenic as the patient did not fit clinical or genetic descriptions of syndromic Microphthalmia type 11 (OMIM, 604294), which is an autosomal recessive disorder with prominent microphtalmia (not seen in our patient) or Chediak-Higashi syndrome (OMIM, 606897), a primary immunodeficiency with partial albinism, leaving the *de novo* mutation in *ASXL3* as the highest candidate disease causing mutation. Of note, analysis of *ASXL1* revealed two non-pathogenic inherited variants and zero *de novo* mutations, ruling out Bohring-Optiz syndrome. The *ASXL3* mutation was confirmed by capillary sequencing in a clinical lab prior to reporting to the family (Additional file 1: Figure S2).

To better understand the rare, nonsynonymous variant burden and impact of mutations in the *ASXL3* gene, we examined the number of variants of a frequency of less than 1% in the *ASXL3* gene in the CPGM internal variant database [19,20]. This database collates every variant detected at the CPGM including the frequency of occurrence and associated clinical phenotypes. The database contains children with suspected rare genetic disorders as well as healthy unaffected family members. In the more than 1,300 exomes sequenced at the CPGM, the proband is the only patient with a heterozygous frameshift mutation in *ASXL3*, predicting a truncated gene product. However, there are 29 single nucleotide variants of with a frequency of less than 1 percent in our database (Additional file 1: Table S1). Characterization of these 29 rare variants revealed 19 that were predicted to deleterious by SIFT (Sorts Intolerant From Tolerant substitutions) [24] and 11 predicted to be probably or possibly damaging by PolyPhen2 (Polymorphism Phenotyping 2) [25]. Phenotypic evaluation of the samples with these rare, non-synonymous variants revealed no patients with developmental delay, microcephaly, or other craniofacial anomalies, suggesting that these variants are unlikely to be pathogenic in a heterozygous state.

## Discussion

In this study we provide the second report and fifth patient with pathogenic mutations in *ASXL3*. Our case provides additional evidence that, indeed, truncating frameshift mutations in the *ASXL3* gene are the cause of a newly recognized distinct disorder characterized by global developmental delay and craniofacial anomalies that shares significant clinical features with Bohring-Opitz syndrome. Our patient, CMH000076, exhibited multiple overlapping clinical features of BOS, most notably trigonocephaly, microcephaly, feeding difficulties, and severe learning difficulties (Table 1). Unlike the previously reported cases with *ASXL3* mutations, our patient had trigonocephaly, thus, the lack of trigoncephaly may not be a useful in differentiating BOS from this newly recognized condition [23]. To date, none of the five patients described with *ASXL3* mutations has displayed the typical BOS-posture, whereas 100 percent of the 30 described patients with *ASXL1* mutations did [14], suggesting that this feature might be useful in discriminating between the two related conditions.

The mutation in our patient was at amino coding position 633, which is closest to subject #3 reported by Bainbridge et al. [23] (659_660del). In contrast to subject #3, however, CMH000079 exhibited a severe phenotype with feeding difficulties, growth restriction and severe global developmental delay. Both the mutation described here and that of the previously described subject #3 occur in an evolutionarily conserved serine-rich motif found between residues ~600-800 [23]. The considerable clinical variability between our patient and that of subject #3 emphasize the need for additional studies of the complex phenotype-genotype associations in this disorder and the paralogous disorder BOS. The diagnosis of HHF1 due to mutation in *ABCC8* in this patient likely contributed to her recurring hospitalizations and growth difficulties, and may have impacted her early development. Importantly, the *ABCC8* variant (c. 2143G > A; p.Val115Met) was also identified by the whole exome sequencing. Labile maternal blood glucose during pregnancy was also considered in the early differential of her developmental delay. However, both the mother and sister of the proband share HHF1 diagnosis and have histories of severe hypoglycemia and with resultant episodes of altered mental status and seizures in early childhood; both have normal cognitive functioning and achieved normal growth parameters.

To date, all reported potential pathogenic mutations in *ASXL3* have been either frameshifting or truncating [23]. To further examine the variant burden in *ASXL3* and the genotype-phenotype relationship, we utilized our clinical grade variant database with more than 1,300 exomes to evaluate rare variation (defined as present in less than 1% of samples) in the *ASXL3* gene. Unlike what has been reported in other large-scale databases, our internal warehouse does not contain samples with truncating, nonsense mutations [23]; however we did uncover 29 rare, single nucleotide variants (SNVs) (Additional file 1: Table S1). *ASXL3* is 6,747 nucleotides long and composed of 12 exons with the 3’ exons 11 and 12 being disproportionately large; comprising 1,957 and 3,708 nucleotides, respectively. The vast majority of the variants from our database were in exon 11, which included our *de novo* deletion or exon 12 (Additional file 1: Table S1). Interestingly, although our database contains 107 samples with neurodevelopmental disorders, none of the discovered rare variants were in any of these patients, suggesting that these variants are unlikely to be pathogenic in a heterozygous state and providing additional support that only frameshifting or truncating mutations in *ASXL3* are pathogenic. However, additional functional studies to investigate the pathogenicity of *ASXL3* variants are needed to completely conclude that SNVs are expected to be nonpathogenic.

## Conclusions

In summary, we describe the use of familial exome sequencing to discover a *de novo* framshift mutation in the *ASXL3* gene in a patient with feeding difficulties, microcephaly, severe global developmental delay, and craniofacial anomalies. Furthermore, we provide additional evidence that heterozygous, frameshift, truncating mutations in *ASXL3* are the cause of a newly recognized disorder [23]. In addition, we expand the knowledge about disease causing mutations and the genotype-phenotype relationships in *ASXL3* and provide evidence that rare, nonsynonymous, and predicted damaging mutations are not associated with developmental delay or microcephaly, rather that pathogenic are likely to be either frameshifting or truncating.

### Consent

The study was approved by the Institutional Review Board of Children’s Mercy Hospital (CMH). Written informed consent was obtained from the parents for publication of this case report and any accompanying images. A copy of the written consent is available for review by the Editor of this journal.

## Abbreviations

ASXL1: Additional sex combs-like 1; ASXL3: Additional sex combs-like 3; BOS: Bohring-Optiz syndrome; CPGM: Center for pediatric genomic medicine; CMH: Children’s mercy hospital; HHF1: Hyperinsulinemic hypoglycemia, familial, type 1; IUGR: Intrauterine growth restriction; MRI: Magnetic resonance imaging; PICC: Peripherally inserted central catheter; PolyPhen2: Polymorphism phenotyping 2; SIFT: Sorts intolerant from tolerant substitutions; SNVs: Single nucleotide variants.

## Competing interests

The authors declare that that they have no competing interests.

## Authors’ contributions

Conceptualized and designed the study SFK. Drafted the initial manuscript: DLD, SES. Coordinated and supervised sample and clinical data collection, obtained consent: SES, LDS. Undertook the exome sequencing: DLD, EGF. Designed the bioinformatics tools, and coordinated bioinformatics efforts: NAM. Carried out the initial analyses: DLD. Reviewed data analysis and oversaw clinical reporting of genetic findings: CJS. Developed clinical-pathological components of bioinformatic tools, and coordinated protocol implementation: NAM, SES. Reviewed and revised the manuscript: SFK, LDS. All authors read and approved the final manuscript.

## Pre-publication history

The pre-publication history for this paper can be accessed here:

http://www.biomedcentral.com/1755-8794/6/32/prepub

## Supplementary Material

Additional file 1 ** Methods. Figure S1.** Next-generation sequencing results viewed in the Integrative Genome Viewer [12] of the *de novo* frameshifting mutation 1897_1898delCA, p.Gln633ValfsX13 in CMH000079 and not present in mother (CMH000080) or father (CMH000081). **Figure S2.** Capillary sequencing results of the *de novo* frameshifting mutation 1897_1898delCA, p.Gln633ValfsX13 in patient with forward and reverse PCR primers and control sample. **Table S1.** Variants at a frequency of less than 1% in *ASXL3* more than 1,300 exomes sequenced at the Center for Pediatric Genomic Medicine at Children’s Mercy Hospital. Click here for file
