# Supplementary material for: De novo frameshift mutation in ASXL3 in a patient with global developmental delay, microcephaly, and craniofacial anomalies
Source: BMC Med Genomics. 2013 Sep 17;6:32. doi: 10.1186/1755-8794-6-32 (PMC3851682; doi:10.1186/1755-8794-6-32)
Supplement: Additional file 1 — Methods. Figure S1. Next-generation sequencing results viewed in the Integrative Genome Viewer [12] of the de novo frameshifting mutation 1897_1898delCA, p.Gln633ValfsX13 in CMH000079 and not present in mother (CMH000080) or father (CMH000081). Figure S2. Capillary sequencing results of the de novo frameshifting mutation 1897_1898delCA, p.Gln633ValfsX13 in patient with forward and reverse PCR primers and control sample. Table S1. Variants at a frequency of less than 1% in ASXL3 more than 1,300 exomes sequenced at the Center for Pediatric Genomic Medicine at Children’s Mercy Hospital. [file 1755-8794-6-32-S1.docx]

ASXL3 Supplement

Methods

**Targeted Exome Sequencing**

Exome sequencing was performed by the Center for Pediatric Genomic Medicine (CPGM) at CMH under a research protocol. Isolated genomic DNA was prepared for sequencing using the Kapa Biosystems library preparation kit and 8 cycles of PCR amplification. Exome enrichment was conducted with the Illumina TruSeq Exome v1 kit (62.2 megabases, 20,794 genes, 201,121 exons) following a slightly modified version of the manufacturer recommended protocol. The enrichment protocol was modified to use the Kapa Biosystems PCR amplification kit for the post-enrichment amplification step to limit polymerase induced GC-bias [[1](#_ENREF_1)]. Successful enrichment was verified by qPCR of 4 targeted loci and 2 non targeted loci of the sequencing library pre- and post-enrichment prior to sequencing [[2](#_ENREF_2)]. The enriched library was sequenced on an Illumina HiSeq 2000 using v3 reagents and 2x101 base pair sequencing reads.

**Next Generation Sequencing Analysis**

Sequence data was generated with Illumina RTA 1.12.4.2 & CASAVA-1.8.2, aligned to the human reference NCBI 37 using the Genomic Short-read Nucleotide Alignment Program (GSNAP) [[3](#_ENREF_3)] and variants were detected and genotyped using the Genome Analysis Toolkit (GATK) [[4](#_ENREF_4)]. Sequence analysis employed FASTQ files, the compressed binary version of the Sequence Alignment/Map format (bam, a representation of nucleotide sequence alignments) and Variant Call Format (VCF, a format for nucleotide variants). Variants were characterized with the CPGM’s Rapid Understanding of Nucleotide variant Effect Software (RUNES v1.0) [[5](#_ENREF_5)]. RUNES incorporates data from the Variant Effect Predictor (VEP) software [[6](#_ENREF_6)], and produces comparisons to NCBI dbSNP, known disease mutations from the Human Gene Mutation Database [[7](#_ENREF_7)] and performs additional *in silico* prediction of variant consequences using ENSEMBL and UCSC gene annotations [[8](#_ENREF_8), [9](#_ENREF_9)]. RUNES categorizes each variant according to the American College of Medical Genetics (ACMG’s) recommendations for reporting sequence variation [[10](#_ENREF_10), [11](#_ENREF_11)] as well as an allele frequency derived from CPGM’s Variant Warehouse database [[5](#_ENREF_5)]. Briefly, category 1 variants are those previously described as disease causing, category 2 are those variants of the type likely to disrupt protein function and be disease causing if they are in a gene associated with disease, and category 3 are variants of unknown significance that may or may not cause disease.

**Capillary Sequencing**

Primers and PCR conditions are available upon request. PCR products were purified using Exo-Sapit (USB Corporation, Cleveland, OH) according to manufacturer’s instructions. Both the forward and reverse strands of the purified PCR product were sequenced using fluorescent dye-terminator sequencing. Sequencing reactions were purified using the BigDye XTerminator Purification Kit (Applied Biosystems, Foster City, CA) according to the manufacturers’ instructions. Results were analyzed on an ABI 3130 analyzer (Applied Biosystems, Foster City, CA). Sequence results were compared to published reference sequence (NM_030632.1) using Sequencher 4.5 (Gene Codes Corporation, Ann Arbor. MI).

Supplement Figure 1. Next-generation sequencing results viewed in the Integrative Genome Viewer [[12](#_ENREF_12)] of the *de novo* frameshifting mutation 1897_1898delCA, p.Gln633ValfsX13 in CMH000079 and not present in mother (CMH000080) or father (CMH000081).


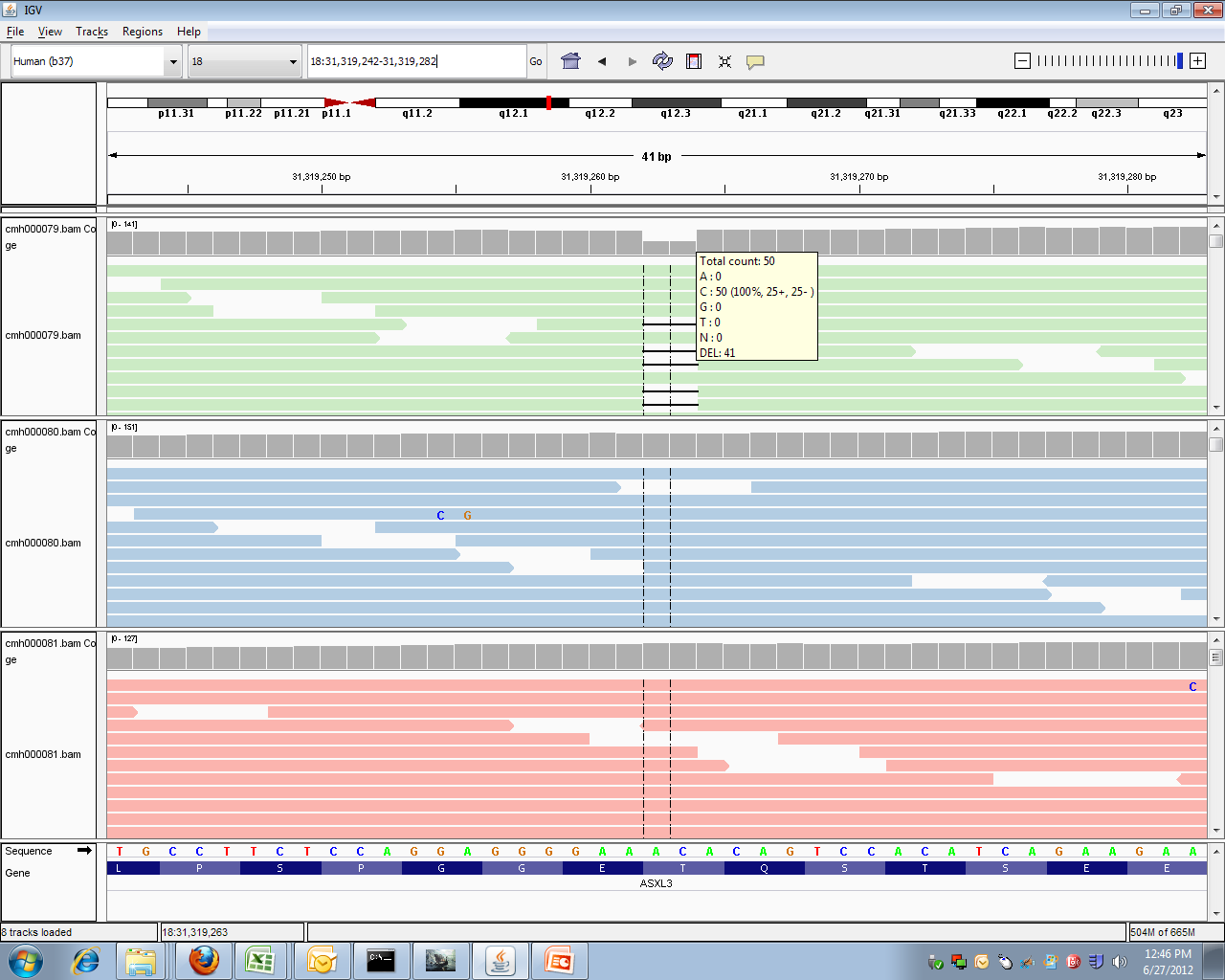


Supplement Figure 2. Capillary sequencing results of the *de novo* frameshifting mutation 1897_1898delCA, p.Gln633ValfsX13 in patient with forward and reverse PCR primers and control sample.


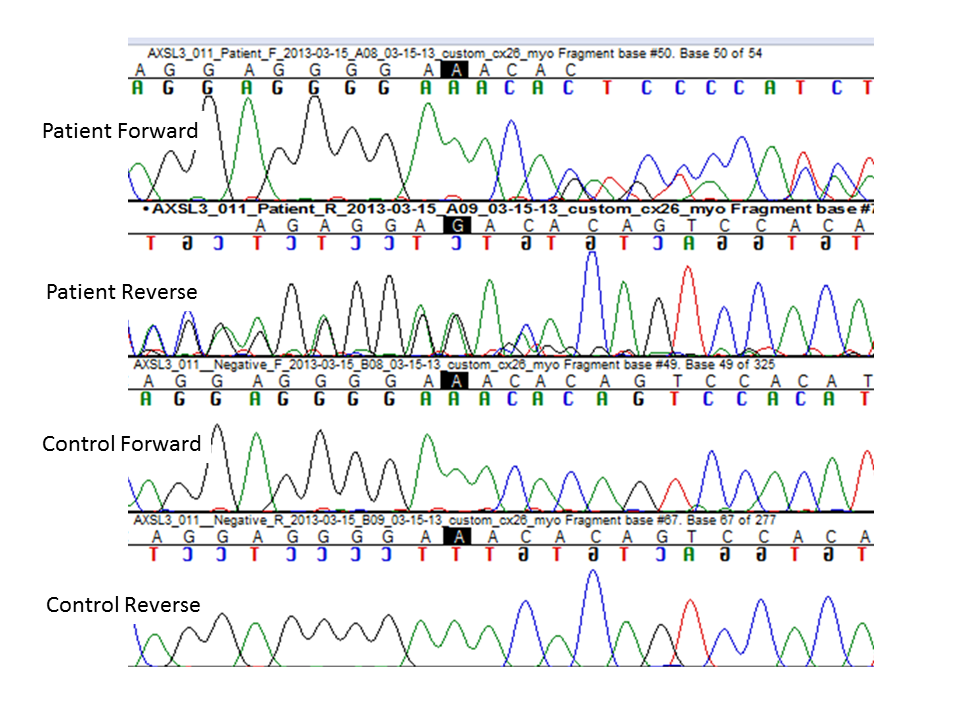


Supplement Table 1. Variants at a frequency of less than 1% in *ASXL3* more than 1,300 exomes sequenced at the Center for Pediatric Genomic Medicine at Children’s Mercy Hospital.

| #chr | start | stop | exon | ref_allele | var_allele | gene | transcript | hgvs_c | translation impact | sift | polyphen | RUNES classification | CMH maf |
| --- | --- | --- | --- | --- | --- | --- | --- | --- | --- | --- | --- | --- | --- |
| 18 | 31319262 | 31319263 | 11 | AC | - | ASXL3 | NM_030632.1 | NM_030632.1:c.1894_1895del | frameshift |  |  | 2 | 0.000502 |
| 18 | 31224959 | 31224959 | 3 | C | A | ASXL3 | NM_030632.1 | NM_030632.1:c.239C>A | non_synonymous | deleterious | probably_damaging | 3 | 0.000502 |
| 18 | 31318588 | 31318588 | 11 | G | C | ASXL3 | NM_030632.1 | NM_030632.1:c.1220G>C | non_synonymous | tolerated | benign | 3 | 0.001004 |
| 18 | 31318777 | 31318777 | 11 | A | C | ASXL3 | NM_030632.1 | NM_030632.1:c.1409A>C | non_synonymous | tolerated | benign | 3 | 0.00251 |
| 18 | 31319287 | 31319287 | 11 | G | A | ASXL3 | NM_030632.1 | NM_030632.1:c.1919G>A | non_synonymous | tolerated | benign | 3 | 0.000502 |
| 18 | 31319592 | 31319592 | 11 | T | G | ASXL3 | NM_030632.1 | NM_030632.1:c.2224T>G | non_synonymous | tolerated | benign | 3 | 0.000502 |
| 18 | 31319710 | 31319710 | 11 | C | T | ASXL3 | NM_030632.1 | NM_030632.1:c.2342C>T | non_synonymous | deleterious | benign | 3 | 0.001004 |
| 18 | 31319905 | 31319905 | 11 | A | G | ASXL3 | NM_030632.1 | NM_030632.1:c.2537A>G | non_synonymous | deleterious | benign | 3 | 0.002008 |
| 18 | 31319947 | 31319947 | 11 | T | A | ASXL3 | NM_030632.1 | NM_030632.1:c.2579T>A | non_synonymous | deleterious | benign | 3 | 0.000502 |
| 18 | 31319970 | 31319970 | 11 | G | A | ASXL3 | NM_030632.1 | NM_030632.1:c.2602G>A | non_synonymous | tolerated | benign | 3 | 0.000502 |
| 18 | 31320099 | 31320099 | 11 | G | A | ASXL3 | NM_030632.1 | NM_030632.1:c.2731G>A | non_synonymous | deleterious | benign | 3 | 0.001506 |
| 18 | 31320321 | 31320321 | 11 | G | C | ASXL3 | NM_030632.1 | NM_030632.1:c.2953G>C | non_synonymous | deleterious | probably_damaging | 3 | 0.000502 |
| 18 | 31320322 | 31320322 | 11 | A | T | ASXL3 | NM_030632.1 | NM_030632.1:c.2954A>T | non_synonymous | deleterious | probably_damaging | 3 | 0.000502 |
| 18 | 31320333 | 31320333 | 11 | C | G | ASXL3 | NM_030632.1 | NM_030632.1:c.2965C>G | non_synonymous | deleterious | benign | 3 | 0.00251 |
| 18 | 31320372 | 31320372 | 11 | C | A | ASXL3 | NM_030632.1 | NM_030632.1:c.3004C>A | non_synonymous | deleterious | probably_damaging | 3 | 0.000502 |
| 18 | 31322993 | 31322993 | 12 | G | A | ASXL3 | NM_030632.1 | NM_030632.1:c.3181G>A | non_synonymous | deleterious | probably_damaging | 3 | 0.000502 |
| 18 | 31323201 | 31323201 | 12 | C | G | ASXL3 | NM_030632.1 | NM_030632.1:c.3389C>G | non_synonymous | tolerated | possibly_damaging | 3 | 0.001506 |
| 18 | 31323808 | 31323808 | 12 | C | G | ASXL3 | NM_030632.1 | NM_030632.1:c.3996C>G | non_synonymous | deleterious | benign | 3 | 0.000502 |
| 18 | 31323916 | 31323916 | 12 | A | C | ASXL3 | NM_030632.1 | NM_030632.1:c.4104A>C | non_synonymous | deleterious | benign | 3 | 0.000502 |
| 18 | 31324035 | 31324035 | 12 | T | C | ASXL3 | NM_030632.1 | NM_030632.1:c.4223T>C | non_synonymous | tolerated | benign | 3 | 0.000502 |
| 18 | 31324052 | 31324052 | 12 | G | A | ASXL3 | NM_030632.1 | NM_030632.1:c.4240G>A | non_synonymous | tolerated | benign | 3 | 0.000502 |
| 18 | 31324056 | 31324056 | 12 | T | G | ASXL3 | NM_030632.1 | NM_030632.1:c.4244T>G | non_synonymous | deleterious | benign | 3 | 0.001004 |
| 18 | 31324143 | 31324143 | 12 | G | A | ASXL3 | NM_030632.1 | NM_030632.1:c.4331G>A | non_synonymous | tolerated | benign | 3 | 0.000502 |
| 18 | 31324401 | 31324401 | 12 | A | G | ASXL3 | NM_030632.1 | NM_030632.1:c.4589A>G | non_synonymous | tolerated | benign | 3 | 0.000502 |
| 18 | 31324419 | 31324419 | 12 | G | T | ASXL3 | NM_030632.1 | NM_030632.1:c.4607G>T | non_synonymous | deleterious | benign | 3 | 0.000502 |
| 18 | 31324629 | 31324629 | 12 | G | A | ASXL3 | NM_030632.1 | NM_030632.1:c.4817G>A | non_synonymous | deleterious | possibly_damaging | 3 | 0.000502 |
| 18 | 31324985 | 31324985 | 12 | G | A | ASXL3 | NM_030632.1 | NM_030632.1:c.5173G>A | non_synonymous | deleterious | possibly_damaging | 3 | 0.000502 |
| 18 | 31326158 | 31326158 | 12 | G | A | ASXL3 | NM_030632.1 | NM_030632.1:c.6346G>A | non_synonymous | deleterious | possibly_damaging | 3 | 0.001506 |
| 18 | 31326213 | 31326213 | 12 | T | G | ASXL3 | NM_030632.1 | NM_030632.1:c.6401T>G | non_synonymous | deleterious | probably_damaging | 3 | 0.001004 |
| 18 | 31326376 | 31326376 | 12 | G | T | ASXL3 | NM_030632.1 | NM_030632.1:c.6564G>T | non_synonymous | deleterious | probably_damaging | 3 | 0.000502 |

Supplement References

1. Quail MA, Otto TD, Gu Y, Harris SR, Skelly TF, McQuillan JA, Swerdlow HP, Oyola SO: **Optimal enzymes for amplifying sequencing libraries**. *Nat Methods* 2012, **9**(1):10-11.

2. Bell CJ, Dinwiddie DL, Miller NA, Hateley SL, Ganusova EE, Mudge J, Langley RJ, Zhang L, Lee CC, Schilkey FD *et al*: **Carrier testing for severe childhood recessive diseases by next-generation sequencing**. *Sci Transl Med* 2011, **3**(65):65ra64.

3. Wu TD, Nacu S: **Fast and SNP-tolerant detection of complex variants and splicing in short reads**. *Bioinformatics* 2010, **26**(7):873-881.

4. DePristo MA, Banks E, Poplin R, Garimella KV, Maguire JR, Hartl C, Philippakis AA, del Angel G, Rivas MA, Hanna M *et al*: **A framework for variation discovery and genotyping using next-generation DNA sequencing data**. *Nat Genet* 2011, **43**(5):491-498.

5. Saunders CJ, Miller NA, Soden SE, Dinwiddie DL, Noll A, Alnadi NA, Andraws N, Patterson ML, Krivohlavek LA, Fellis J *et al*: **Rapid whole-genome sequencing for genetic disease diagnosis in neonatal intensive care units**. *Sci Transl Med* 2012, **4**(154):154ra135.

6. McLaren W, Pritchard B, Rios D, Chen Y, Flicek P, Cunningham F: **Deriving the consequences of genomic variants with the Ensembl API and SNP Effect Predictor**. *Bioinformatics* 2010, **26**(16):2069-2070.

7. Stenson PD, Ball EV, Howells K, Phillips AD, Mort M, Cooper DN: **The Human Gene Mutation Database: providing a comprehensive central mutation database for molecular diagnostics and personalized genomics**. *Hum Genomics* 2009, **4**(2):69-72.

8. Flicek P, Amode MR, Barrell D, Beal K, Brent S, Carvalho-Silva D, Clapham P, Coates G, Fairley S, Fitzgerald S *et al*: **Ensembl 2012**. *Nucleic acids research* 2012, **40**(Database issue):D84-90.

9. Dreszer TR, Karolchik D, Zweig AS, Hinrichs AS, Raney BJ, Kuhn RM, Meyer LR, Wong M, Sloan CA, Rosenbloom KR *et al*: **The UCSC Genome Browser database: extensions and updates 2011**. *Nucleic acids research* 2012, **40**(Database issue):D918-923.

10. Maddalena A, Bale S, Das S, Grody W, Richards S: **Technical standards and guidelines: molecular genetic testing for ultra-rare disorders**. *Genet Med* 2005, **7**(8):571-583.

11. Richards CS, Bale S, Bellissimo DB, Das S, Grody WW, Hegde MR, Lyon E, Ward BE: **ACMG recommendations for standards for interpretation and reporting of sequence variations: Revisions 2007**. *Genet Med* 2008, **10**(4):294-300.

12. Robinson JT, Thorvaldsdottir H, Winckler W, Guttman M, Lander ES, Getz G, Mesirov JP: **Integrative genomics viewer**. *Nat Biotechnol* 2011, **29**(1):24-26.
